# Impact of impaired fractional flow reserve after coronary interventions on outcomes: a systematic review and meta-analysis

**DOI:** 10.1186/s12872-016-0355-7

**Published:** 2016-09-08

**Authors:** Mathias Wolfrum, Gregor Fahrni, Giovanni Luigi de Maria, Guido Knapp, Nick Curzen, Rajesh K. Kharbanda, Georg M. Fröhlich, Adrian P. Banning

**Affiliations:** 1 Oxford Heart Centre, Oxford University Hospitals, Headley Way, Oxford, OX39DU UK; 2 Department of Statistics, TU University Dortmund, Dortmund, Germany; 3 University Hospital Southampton NHS Foundation Trust, Southampton, UK; 4 Department of Cardiology, Charité Universitätsmedizin Berlin (Campus Benjamin Franklin), Berlin, Germany

**Keywords:** Coronary artery disease, Percutaneous coronary interventions, Fractional flow reserve, Intracoronary imaging, Outcome, Meta-analysis

## Abstract

**Background:**

FFR is routinely used to guide percutaneous coronary interventions (PCI). Visual assessment of the angiographic result after PCI has limited efficacy. Even when the angiographic result seems satisfactory FFR after a PCI might be useful for identifying patients with a suboptimal interventional result and higher risk for poor clinical outcome who might benefit from additional procedures. The aim of this meta-analysis was to investigate available data of studies that examined clinical outcomes of patients with impaired vs. satisfactory fractional flow reserve (FFR) after percutaneous coronary interventions (PCI).

**Methods:**

This meta-analysis was carried out according to the Cochrane Handbook for Systematic Reviews. The Mantel-Haenszel method using the fixed-effect meta-analysis model was used for combining the results. Studies were identified by searching the literature through mid-January, 2016, using the following search terms: fractional flow reserve, coronary circulation, after, percutaneous coronary intervention, balloon angioplasty, stent implantation, and stenting. Primary endpoint was the rate of major adverse cardiac events (MACE). Secondary endpoints included rates of death, myocardial infarction (MI), repeated revascularisation.

**Results:**

Eight relevant studies were found including a total of 1337 patients. Of those, 492 (36.8 %) had an impaired FFR after PCI, and 853 (63.2 %) had a satisfactory FFR after PCI. Odds ratios indicated that a low FFR following PCI was associated with an impaired outcome: major adverse cardiac events (MACE, OR: 4.95, 95 % confidence interval [CI]: 3.39–7.22, *p <*0.001); death (OR: 3.23, 95 % CI: 1.19–8.76, *p* = 0.022); myocardial infarction (OR: 13.83, 95 % CI: 4.75–40.24, *p* <0.0001) and repeated revascularisation (OR: 4.42, 95 % CI: 2.73–7.15, *p* <0.0001).

**Conclusions:**

Compared to a satisfactory FFR, a persistently low FFR following PCI is associated with a worse clinical outcome. Prospective studies are needed to identify underlying causes, determine an optimal threshold for post-PCI FFR, and clarify whether simple additional procedures can influence the post-PCI FFR and clinical outcome.

**Electronic supplementary material:**

The online version of this article (doi:10.1186/s12872-016-0355-7) contains supplementary material, which is available to authorized users.

## Background

Fractional flow reserve (FFR) is the established gold standard used in the cardiac catheterisation laboratory to assess the ischemic burden associated with an atheromatous lesion of the coronary arteries. Evidence from various clinical scenarios has shown that an FFR-guided PCI strategy reduces the need for stenting and improves clinical outcomes. Therefore, FFR has been incorporated in current revascularisation guidelines [1–3]. With improved wire technology, it is increasingly cost effective and time-saving to use the pressure wire for diagnosis and as the platform for any subsequent PCI [4]. Nevertheless, post-PCI FFR measurement has not yet become part of established clinical practice and only a minority of operators (22 %) consider FFR to evaluate the post-stenting result [5].

Visual assessment, by angiography and quantitative coronary angiography (QCA), has limited efficacy with respect to identifying patients with suboptimal PCI results and subsequent worse clinical outcomes [6, 7]. Even with an angiographic satisfactory result after PCI 19–32 % of these patients experience an adverse cardiovascular event during a 2-year follow-up [8]. Post-PCI FFR measurement might be a useful indicator for the identification of a suboptimal PCI result and, if so, would be beneficial to both operator and patient. Of note, a recent small prospective interventional study provides evidence that the post-PCI FFR helps to identify patients that might benefit from further optimisation procedures in order to improve clinical outcome [9].

However, the value of post-PCI FFR might be confounded by several factors, such as gender [10] and comorbidities [11]. The subsequent cut-off for a satisfactory post-PCI FFR might differ among patient populations. As the clinical impact of post-PCI FFR has not been determined in larger scale trials this meta-analysis was setup to examine existing data pertaining to post-PCI FFR measurement and its association with clinical outcome.

## Methods

This study was carried according to current recommendations of the Cochrane Handbook for Systematic Reviews and the Meta-analysis of Observational Studies in Epidemiology recommendations (MOOSE checklist, Additional file 1: Table S1) [12]. Two authors (MW, GF) planned and designed this meta-analysis evaluating the association between FFR post PCI and clinical outcomes and created an electronic database with variables of interest.

### Search strategy

Medline, BIOS, and ISI Web of Science databases were searched through January 14, 2016. Additionally, editorials and web-based information sources (http://www.tctmd.com, http://www.theheart.org, http://www.europcronline.com, http://www.cardiosource.com, and http://www.crtonline.com) were screened. The following search terms were used: fractional flow reserve, coronary circulation, after, percutaneous coronary intervention, balloon angioplasty, stent implantation, and stenting. Reference lists of the selected articles were checked for other relevant citations. A more detailed search strategy for Medline can be found in the Additional file 1: Table S2.

### Study selection

Studies included in the meta-analysis were published in full text and in English. Only studies where it was possible to clearly categorise patients into groups with low and high post-PCI FFR were included. Two authors (MW, GF) independently identified appropriate articles. Disagreements were discussed, and a third author (GMF) was consulted in unclear cases. All included studies were approved by the local ethics committees and were in compliance with the Helsinki Declaration.

### Data extraction and quality assessment

Relevant information from each study, retrieved using a dedicated standardised database, included study design, baseline clinical characteristics of the study population, and clinical outcomes. Study quality was ascertained according to the Cochrane Handbook [12], but without using a quality score, due to the limitations associated with this approach [13].

### Endpoints and definitions

Primary endpoint was the rate of major adverse cardiac events (MACE) at longest follow-up. MACE was defined according to the individual study (Table 1). Secondary endpoints included rates of death, myocardial infarction (MI), repeated revascularisation (repeated PCI, target vessel revascularisation [TVR], target lesion revascularisation [TLR] and CABG) and in-stent restenosis.

**Table 1:** Characteristics of included studies

First author, year of publication	Design	Indication for PCI	PCI technique	Cut-off for low FFR - group	FFR technique		Definition of MACE
					Adenosine	Pressure wire pullback	
Bech *et al.,* [18]	Retrospective	Stable angina	POBA	<0.9	i.v.	NA	MACE (death, MI, recurrent angina, CABG, repeated PTCA)
Pijls *et al.,* [31]	Prospective, observational	All comers	“stent” (type NA)	≤ 0.9	i.v. or i.c.	No	MACE (death, MI, CABG, TVR)
Klauss *et al.,* [30]	Retrospective	Stable angina	BMS	< 0.95	i.c.	Not mandatory	MACE (death, MI, TVR)
Nam *et al.,* [19]	Retrospective	2/3 ACS, 1/3 stable angina	DES	≤ 0.9	i.c.	No	MACE (death, MI, TVR)
Leesar *et al.,* [9]	Prospective, interventional	Stable angina	DES > BMS	< 0.96	i.c.	No	MACE (death, MI, TLR)
Ito *et al.*, [23]	Retrospective	92 % stable angina, 8 % unstable angina	DES plus IVUS	≤ 0.9	i.c.	No	MACE (cardiac death, MI, TVR, stent thrombosis)
Reith *et al.*, [24]	Prospective, observational	Stable angina	DES > BMS plus OCT	≤ 0.905	i.c.	No	MACE (death, MI, TLR)
Doh *et al.*, [22]	Prospective, observational	1/3 ACS, 2/3 stable angina	DES plus IVUS	< 0.89	i.v. or i.c.	Not mandatory	TVF (death and MI attributed to target vessel, TVR)

*Abbreviations*: *ACS* acute coronary syndrome, *AUC* area under curve, *BMS* bare metal stent, *CABG* coronary artery bypass graft, *CI* confidence interval, *DES* drug eluting stent, *i.c.* intracoronary, *i.v.* intravenous, *IVUS* intravascular ultrasound, *FFR* fractional flow reserve, *FU* follow up, *MACE* major adverse cardiac events, *MI* myocardial infarction, *Mo* months, *N* patient number, *NA* not applicable, *OCT* optical coherence tomography, *TLR* target lesion revascularisation, *TVF* target vessel failure, *TVR* target vessel revascularisation, *PCI* percutaneous coronary intervention, *POBA* plain old balloon angioplasty

### Data synthesis and analysis

Odds ratio and 95 % confidence intervals (CIs) for binary outcomes were calculated. Since we mostly deal with rare events, Mantel-Haenszel method was used for combining the results [14]. The method can include single-zero and double-zeroes studies. The fixed-effect meta-analysis model was used as no relevant between-study variance was observed (see Additional file 1: Table S3). Weighted incidence of events is presented for both groups calculated according to random-effects meta-analysis for proportions with the Knapp-Hartung adjustment [14, 15]. Given the limitations for the assessment of publication bias in meta-analysis with a small study number using Funnel plots the Egger’s test and Begg’s rank correlation test were applied (Additional file 1: Table S4) [16, 17].

Sensitivity analyses excluded the study by Bech et al. [18], which used only plain old balloon angioplasty (POBA) for the endpoints. Because a post-PCI FFR <0.9 has been identified as an optimal predictor of a worse clinical outcome [19], one analysis considered only studies with an FFR cut-off of 0.9 between low and high FFR groups. All meta-analyses were carried out using the package metafor in the statistical software package R, version 3.2.3 [20, 21].

## Results

### Included studies

**Fig. 1:**
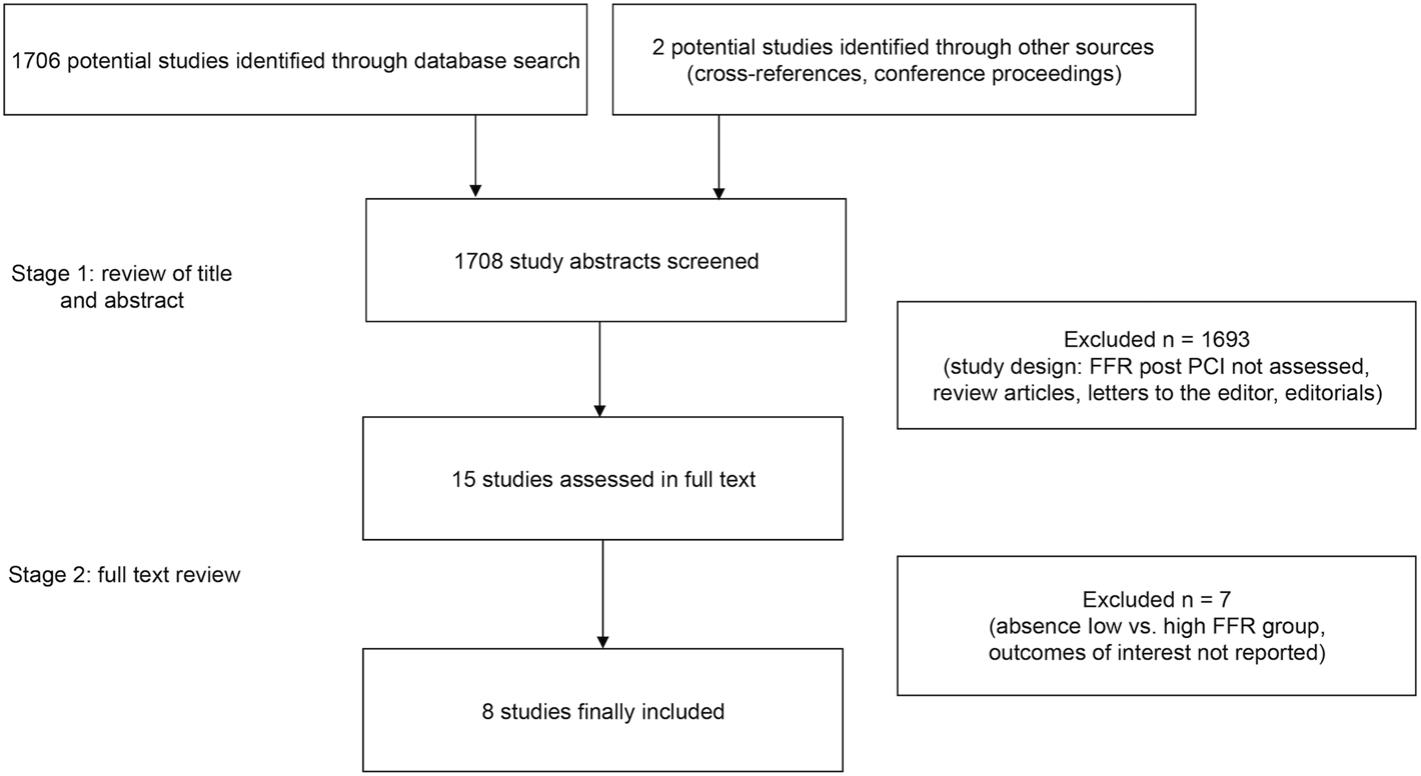
Study selection process. FFR - fractional flow reserve, PCI - percutaneous coronary intervention

A total of eight studies with 1337 patients met our inclusion criteria: four prospective studies (983 patients) and four retrospective studies (354 patients), published between 1999 and 2015 (Fig. 1). Study and population characteristics are presented in Tables 1 and 2.

**Table 2:** Patient characteristics

	Bech *et al.*		Pijls *et al.*		Klauss *et al.*		Nam *et al.*		Leesar *et al.*		Ito *et al.*		Reith *et al.*		Doh *et al.*		Combined	
FFR group	Low	High	Low	High	Low	High	Low	High	Low	High	Low	High	Low	High	Low	High	Low	High
*N*	32	26	237	507	53	66	40	40	31	35	53	44	26	40	20	95	492	853
Age (years) (SD)	61.5		62 (11)		62 (10)		62 (10)	63 (8)	63 (11)	60 (12)	71 (9)		69 (10)	69 (10)	64 (9)		65 (4)	64 (4)
																	64 (4)	
Male (%)	69		NA		75		78	70	77	70	75		88	75	83		80	77
																	77	
HTN (%)	33		51		79		58	43	68	94	92		65	88	82		63	74
																	61	
Diabetes (%)	17		24		26		20	8	32	23	37		58	53	51		34	28
																	28	
HC (%)	31		61		83		10	15	58	74	85		54	60	68		37	49
																	61	
Smoking (%)	24		48		38		28	48	32	46	54		15	23	30		26	38
																	41	
FHx (%)	41		38		38		NA		NA		NA		39	63	NA		40	
Prior MI (%)	17		NA		56		10	13	NA		NA		NA		5.6		25	
MVD (%)	NA		NA		67		63	60	NA		NA		73	68	63		67	64
																	65	
LAD (%)	66		52		39		83	55	39	34	56		NA		100	71	71	59
																	54	
Complex lesion^a^ (%)	NA		NA		65		90	78	NA		NA		NA		95	67	92	70
																	72	
FU (months)	24		6		6		12		24		18		20		23		16	

Separate data provided for low FFR group and high FFR group if available from respective study, otherwise overall value

*Abbreviations*: *FHx* family history, *FFR* fractional flow reserve, *FU* follow up, *HC* hypercholesterolemia, *HTN* Hypertension, *LAD* left anterior descending artery, *MI* myocardial infarction, *MVD* multi-vessel-disease, *N* patient number, *SD* standard deviation

^a^Complex lesion is defined as B2 or C category according to American College of Cardiology/American Heart Association (ACC/AHA) lesion classification

### Main outcomes

#### MACE

**Fig. 2:**
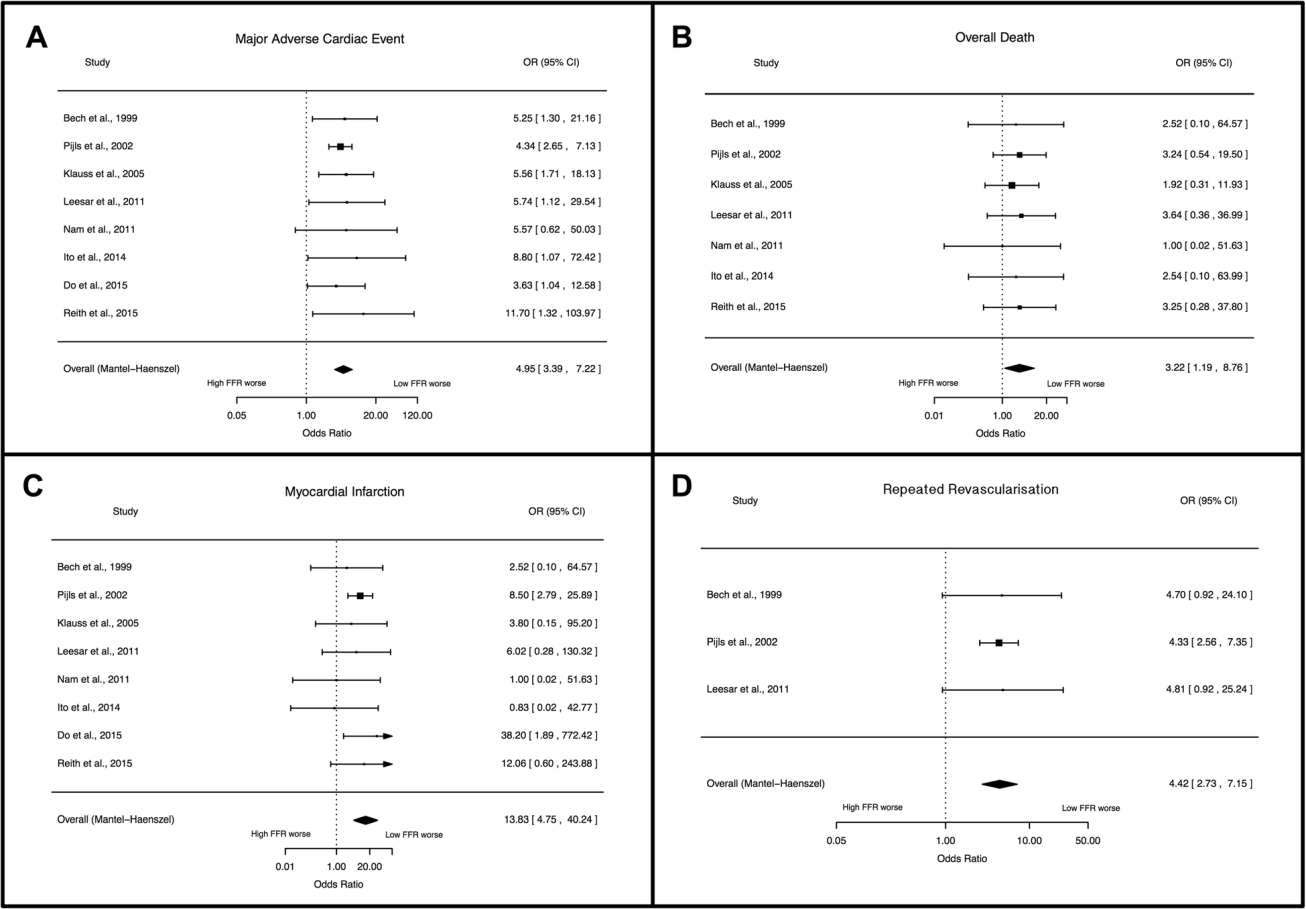
Forest plots of odds ratios (OR) for major adverse cardiac events (MACE, Panel **a**), death (Panel **b**), myocardial infarction (Panel **c**) and repeated revascularisation (Panel **d**). Markers represent point estimates of odds ratios, marker size represents study weight. *Horizontal bars* indicate 95 % confidence intervals (CI). FFR - fractional flow reserve

MACE rate was defined (Table 1) and reported by all included studies. The overall weighted incidence for MACE was 21.4 (95 % CI: 17.0–25.8 %) in the low FFR group and 5.0 (95 % CI: 3.5–6.4 %) in the high FFR group (Fig. 2a). A low FFR after PCI was associated with significantly higher odds for MACE (OR: 4.95, 95 % CI: 3.39–7.22, *p* <0.001).

#### Death

Overall rate of death was reported by seven studies involving 1230 patients. The weighted incidence for overall death was 1.7 % (95 % CI: 0.6–2.9 %) in the low FFR group and 0.8 % (95 % CI: 0.0–1.7 %) in the high FFR group (Fig. 2b). A low FFR after PCI was associated with significantly higher risk of death (OR: 3.23, 95 % CI: 1.19–8.76, *p* = 0.022).

#### Myocardial infarction

The event rate for MI was reported by all included studies. The weighted incidence for MI was 3.3 (95 % CI: 1.1–5.6 %) in the low FFR group and 0.8 % (95 % CI: 0.2–1.4 %) in the high FFR group (Fig. 2c). A low FFR after PCI was associated with significantly higher risk of MI during follow-up (OR: 13.83, 95 % CI: 4.75–40.24, *p* < 0.0001).

#### Repeated revascularisation

Data for repeated revascularisation was available for repeated PCI from three studies, and for CABG from 5 studies. There was an increased risk for repeated revascularisation for patients in the low FFR group, compared to patients in the high FFR group (OR: 4.42, 95 % CI: 2.73–7.15, *p* < 0.0001, Fig. 2d): repeated PCI (OR: 3.81, 95 % CI: 2.26–6.43, *p* < 0.0001), CABG (OR: 6.35, 95 % CI: 1.96–20.54, *p* = 0.002,). Four studies included data about TVR [9, 19, 22, 23], and two studies about TLR [9, 24], and these indicated that the risk for both endpoints was higher for patients with impaired FFR after PCI: TVR (OR: 3.40, 95 % CI: 1.44–8.03, *p* = 0.005), TLR (OR: 5.48, 95 % CI: 1.12–26.78, *p* = 0.036). Two studies reported the rate of in-stent restenosis [9, 19]. The weighted incidence for in-stent restenosis was 16.9 % (95 % CI: 8.2–25.6 %) in the low FFR group and 3.4 % (95 % CI: 0.0–7.5 %) in the high FFR group. A low FFR after PCI was associated with significantly higher risk of in-stent restenosis (OR: 4.93, 95 % CI: 1.32–18.37, *p* = 0.018).

#### Sensitivity analysis

After excluding the study by Bech et al. [18], the results still demonstrated that patients with a low post-PCI FFR had a significantly higher risk for all main outcomes than patients in the high FFR group (Additional file 1: Figure S1): MACE (OR: 4.93, 95 % CI: 3.33–7.29, *p* < 0.0001), death (OR: 3.03, 95 % CI: 1.11–8.28, *p* = 0.03), MI (OR: 13.45, 95 % CI: 4.63–39.05, *p* < 0.0001) and repeated PCI (OR: 3.96, 95 % CI: 2.29–6.85, *p* < 0.0001). When only studies with an FFR cut-off of 0.9 were included (Additional file 1: Figure S2), the results showed that patients in the low FFR group had significantly higher risk than the high FFR group for MACE (OR: 4.92, 95 % CI: 3.11–7.78, *p* < 0.0001), myocardial infarction (OR: 10.02, 95 % CI: 3.33–30.12, *p* < 0.0001), and a statistical trend towards higher risk for overall death (OR: 3.70, 95 % CI: 0.89–15.43, *p* = 0.073). Other endpoints were not included in this analysis, as the number of appropriate studies was ≤ 2.

## Discussion

### Post-PCI FFR as an indicator of clinical outcome

Results of the meta-analysis reported herein, which included different clinical scenarios and data from different era of interventional cardiology, support the hypothesis that a persistently low FFR following PCI is associated with an adverse clinical outcome. We found that patients with an impaired post-PCI FFR had significantly increased risk for the primary endpoint of MACE, as well as for the secondary endpoints of death, myocardial infarction, and repeat revascularization (PCI and CABG). These results were not changed by the exclusion of data from one study that used only POBA [18], a technique known to convey a higher risk for MACE than stenting [25, 26].

Data supporting present findings of the primary endpoint of MACE comes from a study by Johnson et al. [27], which primarily studied the continuous relationship between pre-PCI FFR and clinical outcomes. A small sub-analysis of this study focused on FFR measured immediately after stenting and showed that low FFR is inversely correlated with subsequent adverse events in both continuous (Cox hazard ratio: 0.86, 95 % CI: 0.80–0.93; *P* < 0.001) and tertile (log- rank *P* < 0.001) analyses.

### Causes of persistently low FFR after PCI

**Fig. 3:**
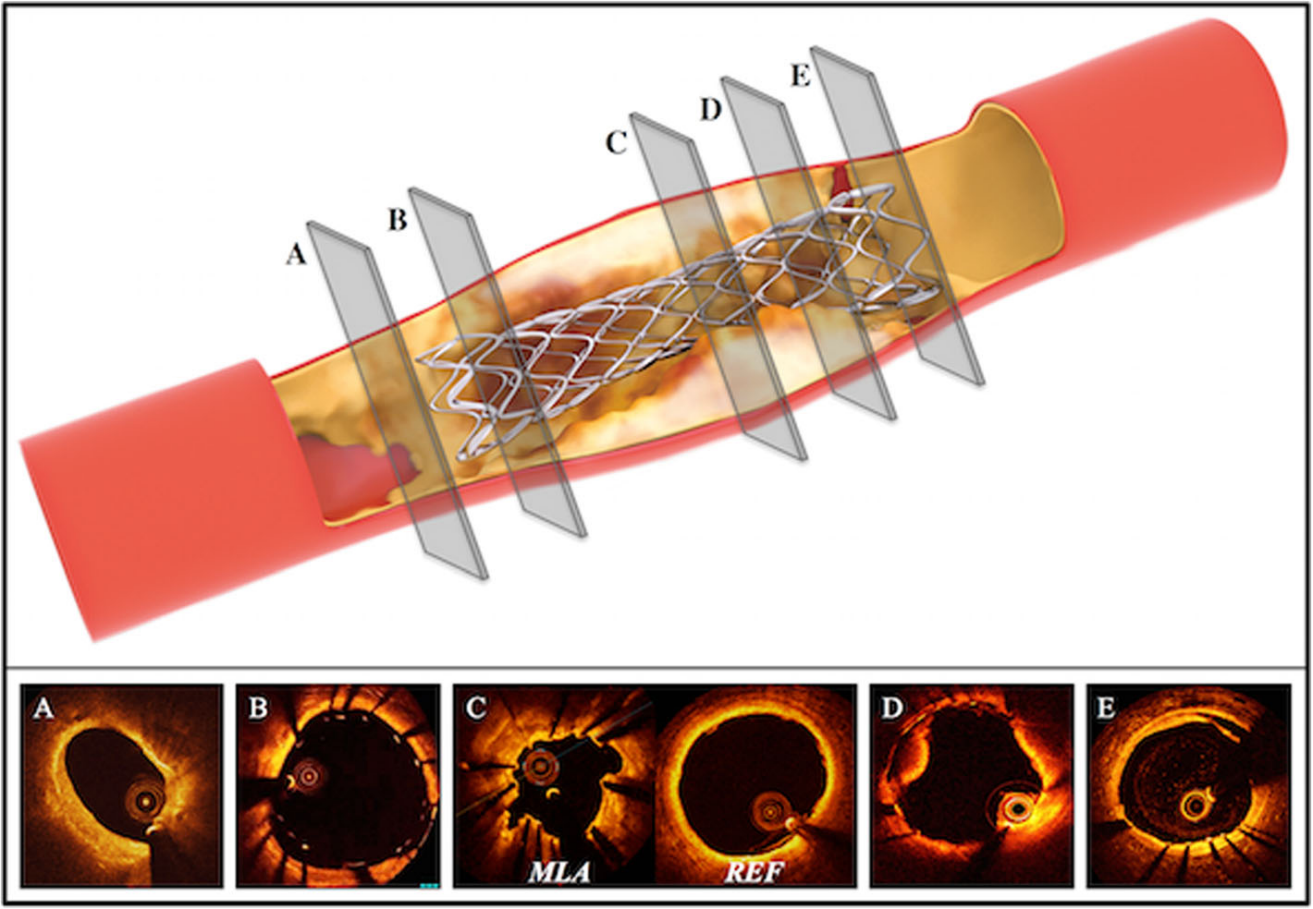
Potential causes of suboptimal FFR after percutaneous coronary interventions. Panel **a** ‘geographical miss’ (diseased reference segment). Panel **b** stent mal-apposition. Panel **c** stent under-expansion. Panel **d** intrastent plaque-protrusion/thrombus. Panel **e** edge dissection. MLA - minimal lumen area, REF - proximal reference segment

A number of factors can cause a post-PCI pressure drop over a treated epicardial segment, eventually leading to an impaired FFR, including incomplete stent expansion, stent malapposition, “geographical miss,” plaque protrusion, edge dissection, and plaque shift at the stent edge (Fig. 3).

The pathophysiological concept is explained by the Hagen-Poiseuille law (e.g. if malapposition or plaque protrusion is present): the pressure loss is caused by viscous friction along a treated segment. Additionally, e.g. if incomplete stent-expansion or geographical miss is considered the Bernoulli’s law can be applied. In this scenario residual narrowing leads to a conversion of pressure into kinetic energy. As a substantial amount of energy is lost due to the presence of turbulent flow not the entire coronary pressure can be recovered at the exit of the target lesion. Even without a significant narrowing a suboptimal PCI result can cause turbulent flow within and beyond the treated coronary segment (stent malapposition, plaque protrusion, edge dissection, plaque shift at the stent edge), especially during the hyperaemic phase of FFR assessment, when high flow rates of blood are induced. The switch from laminar to turbulent flow results in the formation of eddies and a dramatic increase in flow resistance, leading to a pressure drop downstream from the treated coronary segment. The total pressure loss during hyperaemia causes the impaired FFR.

The first study to establish a link between a persistent pressure gradient and a suboptimal stent result was published by Hanekamp et al., who used quantitative coronary angiography, intravascular ultrasound, and coronary pressure measurement to assess deployment of coil stents [28]. They found a close relationship between poor PCI results, such as stent underexpansion and/or malapposition, and suboptimal stent symmetry, and in-stent gradients. Another study using slotted-tube stents found that a post-stent FFR <0.96 predicted a suboptimal stent result identified by IVUS [29]. In 70 % of those patients, a low post-stent FFR was linked to incomplete stent expansion. Studies using drug eluting stents confirmed a substantial rate of under-expanded stents in patients with low post-stent FFR, despite reasonable angiographic results [22, 30]. One of these studies found that low post-stent FFR correlated with adverse clinical outcomes at three years [22].

A number of additional factors can affect the final results of stent implantation and account for the relationship between post-PCI FFR and clinical outcome. Ito et al. suggested a link between high residual plaque volume, identified by IVUS, and impaired post-stent FFR after DES implantation [23]. Patients with high residual plaque had a significantly higher rate of MACE after 18 months.

Lesion complexity may also affect the final result of stent-implantation. Two included studies of our meta-analysis reported a significant lower FFR in patients with complex lesions [19, 22]. In both studies a low FFR post PCI was associated with a significantly higher rates of MACE rates (Table 2). Stent dimensions were also reported to influence post-stent FFR and clinical outcome [19, 22, 31]. Stents with greater length or smaller diameter were significantly correlated with a low post-stent FFR and higher MACE rates. These observations might help to explain the previously described association between these characteristics in BMS and DES procedures and higher subsequent event rates [32–34].

Variations in post-PCI FFR and ultimately outcome seem to be linked to different coronary arteries. A suboptimal post-stent FFR was more frequent in patients with a PCI to the left anterior descending (LAD) artery than to the right coronary artery or left circumflex [19, 22]. This finding presumably reflects the larger myocardial territory subtended by the LAD, leading to greater peak flow and lower post-stent FFR for any given residual stenosis in this vessel. Interestingly, in the aforementioned studies an impaired post-PCI FFR was associated with higher rate of MACE.

### Potential clinical application of the results

Based on the results of our meta-analysis, use of a pressure wire to check the PCI result seems to be a promising concept, even after PCI with an apparently satisfactory angiographic result. Measurement of post-PCI FFR is especially appropriate when a pressure wire was used before PCI to guide the treatment strategy. If FFR remains low after PCI and a pressure wire pullback manoeuvre during maximal hyperaemia demonstrates a step increase of pressure within or close to the stent edges subsequent intravascular imaging might provide the ability to identify causes of suboptimal post-PCI FFR (Fig. 3). Based on the result of imaging, simple additional procedures could improve the interventional result. However, only one small previous study has prospectively examined whether additional interventions can reduce the risk of future MACE in patients with low post-PCI FFR [9]. Their results suggested that consistent postdilatation after coronary stenting in patients with a post-stent FFR ≥0.96 could achieve consequently favourable clinical outcomes in 53 % of patients.

In contrast, a continuous gradual reduction in FFR during pressure wire pullback manoeuvre along the course of the coronary artery suggests diffuse CAD. The observation of diffuse CAD may not be evident from an angiographic examination, but can lead to severely impaired FFR (<0.75), with myocardial ischemia and high rates of MACE [35]. For example, diffuse CAD is more common in patients with diabetes mellitus and, in these patients, is often associated with impaired post-stent FFR, despite an angiographically optimal PCI [36]. Thus, low post-stent FFR without a notable point of decrease could indicate advanced diffuse CAD, associated with a relatively high MACE rate. This is an important finding as further interventional optimisation of PCI is not a promising option for diffuse CAD and should be deferred. Patients with diffuse CAD might benefit from a stronger medical secondary prevention or from prolonged dual antiplatelet therapy. However, at current stage these strategies are only hypothetical and need validation in large scale randomised clinical trials.

### Study limitations

Most of the studies included in the meta-analysis were retrospective or observational and, therefore, were subject to patient selection bias, lack of independent event adjudication, heterogeneity in event definitions, and differences in the duration of follow-up (Tables 1 and 2). With the exception of one large multicenter registry [31], most of the studies had small sample sizes, ranging from 60 to 119 patients. Most studies had no restrictions with regard to co-morbidities, which led to a generally heterogeneous patient population (Table 2).

The existing data on post-PCI FFR covers a wide range of coronary interventions, including POBA, BMS, and first and second generation DES (Table 1). Because different stent designs differentially affect blood flow and post-stent FFR, the results must be considered hypothesis-generating. Furthermore, induction of hyperaemic FFR was accomplished by intracoronary adenosine in the majority of trials, so the results cannot entirely be compared to cases using intravenous adenosine, especially in combination with pressure wire pullback.

Some of the studies included in the meta-analysis used different cut-off points to distinguish between low and high FFR groups. Therefore, we included a sensitivity analysis that considered only studies with an FFR cut-off of 0.9, the value identified as an optimal predictor for a worse clinical outcome following PCI [19]. The results demonstrated a significantly higher risk for patients in the low FFR group for MACE and myocardial infarction, and a trend towards a higher risk for overall death. However, prospective validation of this cut-off is an important future challenge. It is conceivable that the optimal cut-off will vary among different coronary arteries and different clinical scenarios, similar to results of FFR assessment before PCI [27].

Furthermore, current data provides evidence only for the use of conventional FFR. However, alternative modes of physiological assessment are promising such as the instantaneous wave-free ratio (iFR). iFR assess the severity of a coronary stenosis using a pressure wire without the need for potent vasodilators. This might be an advantage over FFR as it obviates the need for adenosine, which is contraindicated in some patients and could safe time and costs in the cathlab. Especially with the introduction of iFR pullback (iFR Scout™, Volcano Corporation, San Diego, California) it seems to be a promising concept for the assessment of the PCI result. However, no data exists on this topic yet and further research is strongly warranted.

## Conclusion

Results of the meta-analysis presented herein provide evidence that a persistently low FFR following PCI is associated with an adverse clinical outcome. Prospective studies are warranted to determine an optimal threshold (or thresholds, in different scenarios) for post-PCI FFR. Observational studies using intra-coronary imaging, such as IVUS and OCT, have suggested different underlying causes for a suboptimal FFR. Large prospective studies are needed to confirm that these are the responsible mechanisms and to examine whether additional simple procedures can influence post-stent FFR and potentially improve clinical outcome.

## Additional file

Additional file 1: This supplement contains additional methods and results. (DOC 856 kb)

## Abbreviations

ACS: Acute coronary syndrome
AUC: Area under curve
BMS: Bare metal stent
CAD: Coronary artery disease
CI: Confidence interval
DES: Drug eluting stent
FFR: Fractional flow reserve
iFR: Instantaneous wave-free ratio
IVUS: Intravascular ultrasound
MACE: Major adverse cardiac events
MSA: Minimal stent area
OCT: Optical coherence tomography
PCI: Percutaneous coronary intervention
POBA: Plain old balloon angioplasty
ROC: Receiver operating characteristic curve
TLR: Target lesion revascularisation
TVF: Target vessel failure
TVR: Target vessel revascularisation
