# Supplementary material for: Impact of impaired fractional flow reserve after coronary interventions on outcomes: a systematic review and meta-analysis
Source: BMC Cardiovasc Disord. 2016 Sep 8;16(1):177. doi: 10.1186/s12872-016-0355-7 (PMC5017064; doi:10.1186/s12872-016-0355-7)
Supplement: Additional file 1: — This supplement contains additional methods and results. (DOC 856 kb) [file 12872_2016_355_MOESM1_ESM.doc]

**SUPPLEMENT TO: Impact of impaired fractional flow reserve after coronary interventions on outcomes: a systematic review and meta-analysis**

This supplement contains additional methods and results.

**SUPPLEMENTAL METHODS**

**Assessment of heterogeneity**

Variance between studies is presented by Tau^2^, which is the estimated variance of underlying effects across studies; heterogeneity of the studies was quantified with Q - statistic (Online Table S2).^1^ A Q < 0.1 was considered significant.

**SUPPLEMENTAL TABLES**

**eTable 1. MOOSE Checklist.**

|  | **Reported on page** | **Comments** |
| --- | --- | --- |
| **Reporting of background should include** | | |
| Problem definition | 4 |  |
| Hypothesis statement | 4 | A persistently low FFR following PCI is associated with a worse clinical outcome. |
| Description of study outcomes | 6 |  |
| Type of exposure or intervention used | 5 |  |
| Type of study designs used | 5 |  |
| Study population |  | No restrictions were used. |
| **Reporting of search strategy should include** | | |
| Qualifications of searchers (eg librarians and investigators) |  | The credentials of the three investigators MW, GF, and GMF are indicated in the author list. |
| Search strategy, including time period used in the synthesis and key words | 5 |  |
| Effort to include all available studies, including contact with authors | 5 |  |
| Databases and registries searched | 5 |  |
| Search software used, name and version, including special features used (eg explosion) |  | No search software was used. Endnote (Version X7.4) was used to merge retrieved citations and eliminate duplications. |
| Use of hand searching (eg reference lists of obtained articles) | 5 |  |
| List of citations located and those excluded, including justification | Figure 1 |  |
| Method of addressing articles published in languages other than English | 5 |  |
| Method of handling abstracts and unpublished studies |  | Unpublished studies were not found on the association. |
| Description of any contact with authors |  | No contact with authors was necessary. |
| **Reporting of methods should include** | | |
| Description of relevance or appropriateness of studies assembled for assessing the hypothesis to be tested | 5 |  |
|  | **Reported on page** | **Comments** |
| Rationale for the selection and coding of data (eg sound clinical principles or convenience) |  | Data extracted from each of the studies were relevant to the population characteristics, study design, exposure, outcome, and possible effect modifiers of the association. |
| Documentation of how data were classified and coded (eg multiple raters, blinding and interrater reliability) | 5 |  |
| Assessment of confounding (eg comparability of cases and controls in studies where appropriate) | 6 | Sensetivity analysis were carried out. |
| Assessment of study quality, including blinding of quality assessors, stratification or regression on possible predictors of study results | 6 |  |
| Assessment of heterogeneity | Supplement Page 1 |  |
| Description of statistical methods (eg complete description of fixed or random effects models, justification of whether the chosen models account for predictors of study results, dose-response models, or cumulative meta-analysis) in sufficient detail to be replicated | 6 |  |
| Provision of appropriate tables and graphics |  | Details regarding the search terms for database search were were included in 1 table, 1 flow chart, 2 summary table, 2 figures with forest plots, 1 figure describing the background. |
| **Reporting of results should include** | | |
| Graphic summarizing individual study estimates and overall estimate |  | Figure 2 |
| Table giving descriptive information for each study included |  | Table 1 and 2 |
| Results of sensitivity testing (eg subgroup analysis) |  | eFigure 1 and 2 |
| Indication of statistical uncertainty of findings |  | 95% confidence intervals were presented with all summary estimates and results of sensitivity analyses. A *p*-value < 0.05 was considered statistically significant |
| **Reporting of discussion should include** | | |
| Quantitative assessment of bias (eg publication bias) | 13 |  |
| Justification for exclusion (eg exclusion of non-English language citations) |  | No non-English language citation with potential data had to be excluded. |
| Assessment of quality of included studies | 13 |  |
|  | **Reported on page** | **Comments** |
| **Reporting of conclusions should include** | | |
| Consideration of alternative explanations for observed results | 12 |  |
| Generalization of the conclusions (eg appropriate for the data presented and within the domain of the literature review) | 14 |  |
| Guidelines for future research | 14 |  |
| Disclosure of funding source |  | No funding source to disclose. |

FFR - fractional flow reserve. PCI - percutaneous coronary intervention.

**eTable 2. Search strategy for MEDLINE (search date January 14, 2016).**

| **Search** | **# of abstracts** |
| --- | --- |

Search History

Limits: no

#8 Search (#1 OR #2) AND #3 AND (#4 OR #5 OR #6 OR #7) 1706

#7 Search “stenting” [All] 23781

#6 Search “stent implantation” [All] 7569

#5 Search “balloon angioplasty” [All] 51188

#4 Search “percutaneous coronary intervention” [All] 22061

#3 Search "after" [All] 3820078

#2 Search "coronary circulation " [All] 35618

#1 Search " fractional flow reserve" [All] 36850

**eTable 3**. Heterogenity test for primary and secondary endpoints.

| **Endpoint** | **Heterogenity** |
| --- | --- |
|
| **MACE** | Tau^2^ =0, Q = 1,4458, p=0,9841 |
| **Death** | Tau ^2^ =0, Q = 0,5017, p = 0,9978 |
| **Myocardial infarction** | Tau^2^ = 0, Q = 4,0490, p = 0,7741 |
| **Repeated PCI** | Tau^2^ = 0, Q = 0,2324, p = 0,8903 |
| **CABG** | Tau^2^ = 0, Q = 0,8799, p = 0,9274 |
| **TVR** | Tau^2^ = 0, Q = 2,2514, p = 0,5219 |
| **TLR** | Tau^2^ = 0, Q = 0,5713, p = 0,4497 |
| **In-stent restenosis** | Tau^2^ = 0, Q = 0,4667, p = 0,4945 |

CABG - coronary artery bypass graft, MACE - major adverse cardiac events, PCI - percutaneous coronary intervention, TLR - target lesion revascularisation, TVR - target vessel revascularisation

**eTable 4.** Assessment of publication bias for the primary endpoint

| **Endpoint** | **Egger’s test** | **Begg’s rank correlation** |
| --- | --- | --- |
| **MACE** | *p* = 0.39 | *p* = 0.11 |

MACE - major adverse cardiac events

**SUPPLEMENTAL FIGURES**

**eFigure 1. Sensitivity analysis excluding plain old balloon angioplasty (POBA).**


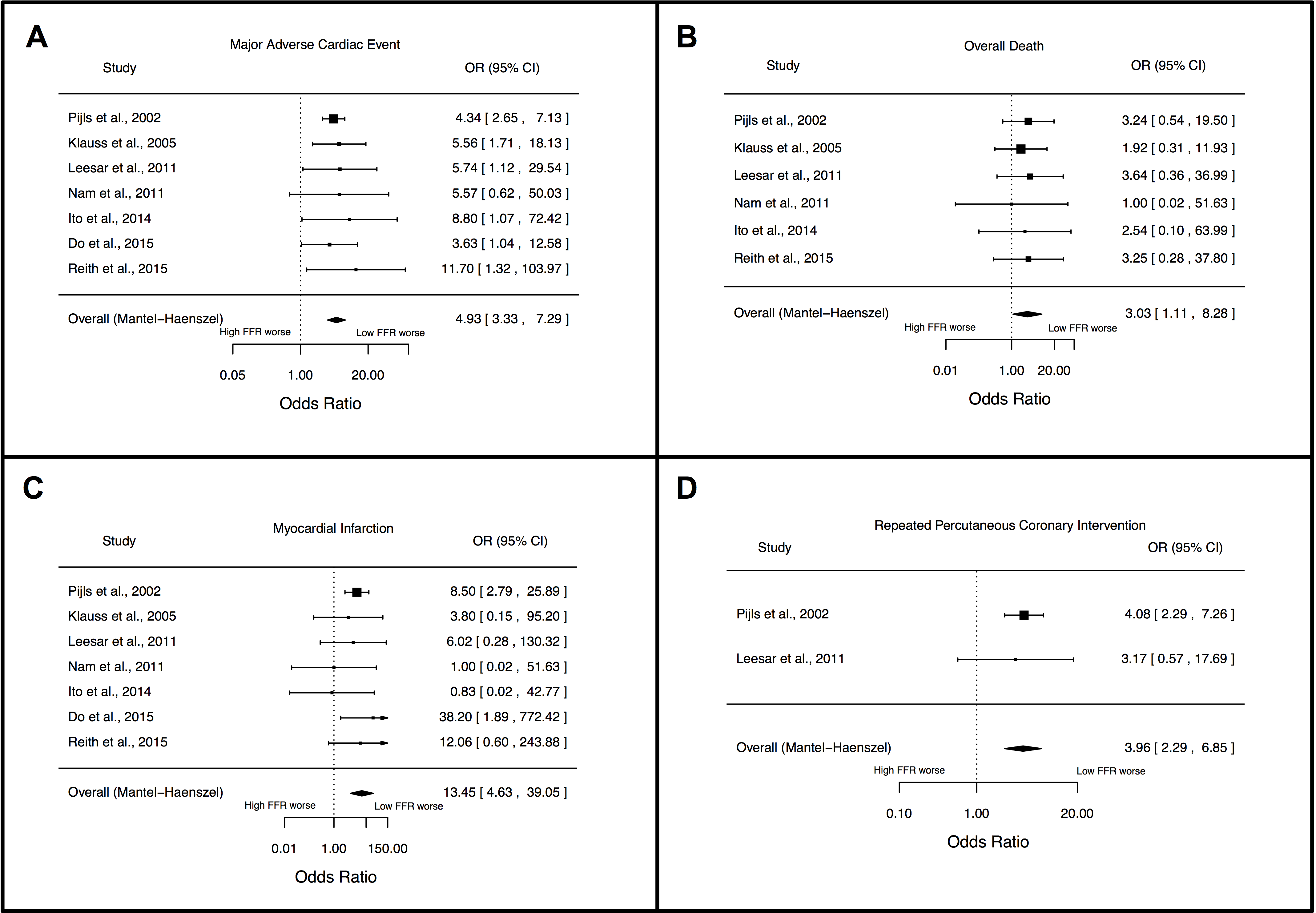


Forest plots of odds ratios (OR) for major adverse cardiac events (MACE, Panel A), death (Panel B), myocardial infarction (Panel C) and repeated percutaneous coronary intervention (Panel D). Markers represent point estimates of odds ratios, marker size represents study weight. Horizontal bars indicate 95% confidence intervals (CI). FFR - fractional flow reserve.

**eFigure 2. Sensitivity analysis considering only studies with an FFR cut-off of 0.9 between low and high FFR groups.**


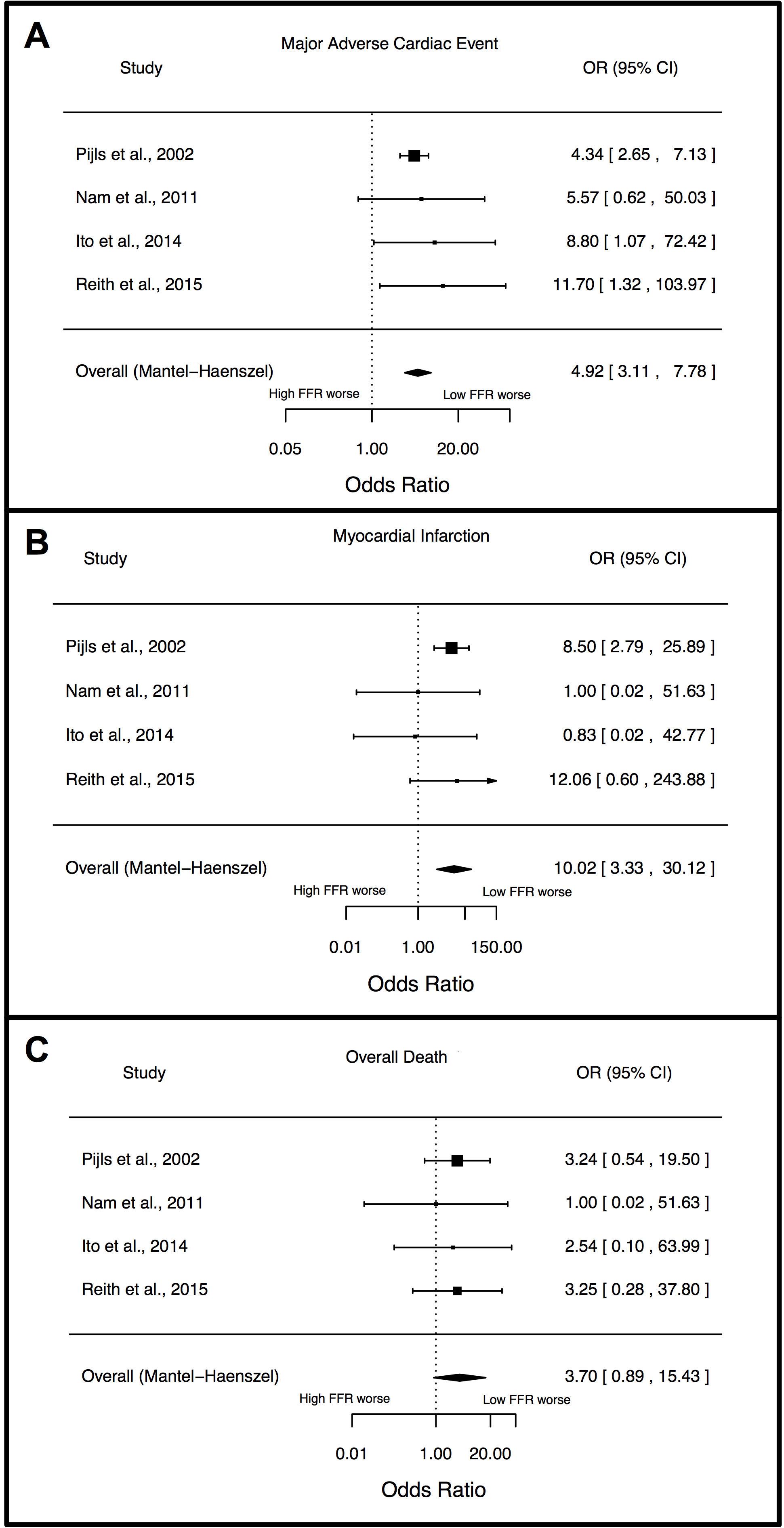
Forest plots of odds ratios (OR) for major adverse cardiac events (MACE, Panel A), death (Panel B) and myocardial infarction (Panel C). Markers represent point estimates of odds ratios, marker size represents study weight. Horizontal bars indicate 95% confidence intervals (CI). FFR - fractional flow reserve.

**SUPPLEMENTAL REFERENCES**

1. Higgins JP, Thompson SG. Quantifying heterogeneity in a meta-analysis. Stat Med 200**2;**21(11):1539-58.
